# The Discriminant Value of Phase-Dependent Local Dynamic Stability of Daily Life Walking in Older Adult Community-Dwelling Fallers and Nonfallers

**DOI:** 10.1155/2015/402596

**Published:** 2015-09-30

**Authors:** Espen A. F. Ihlen, Aner Weiss, Jorunn L. Helbostad, Jeffrey M. Hausdorff

**Affiliations:** ^1^Department of Neuroscience, Norwegian University of Science and Technology, 7491 Trondheim, Norway; ^2^Center for the Study of Movement, Cognition, and Mobility, Department of Neurology, Tel Aviv Sourasky Medical Center, 64239 Tel Aviv, Israel; ^3^Clinic for Clinical Services, St. Olavs Hospital, Trondheim University Hospital, 7006 Trondheim, Norway; ^4^Department of Physical Therapy, Sackler School of Medicine and Sagol School of Neuroscience, Tel Aviv University, Tel Aviv, Israel

## Abstract

The present study compares phase-dependent measures of local dynamic stability of daily life walking with 35 conventional gait features in their ability to discriminate between community-dwelling older fallers and nonfallers. The study reanalyzes 3D-acceleration data of 3-day daily life activity from 39 older people who reported less than 2 falls during one year and 31 who reported two or more falls. Phase-dependent local dynamic stability was defined for initial perturbation at 0%, 20%, 40%, 60%, and 80% of the step cycle. A partial least square discriminant analysis (PLS-DA) was used to compare the discriminant abilities of phase-dependent local dynamic stability with the discriminant abilities of 35 conventional gait features. The phase-dependent local dynamic stability *λ* at 0% and 60% of the step cycle discriminated well between fallers and nonfallers (AUC = 0.83) and was significantly larger (*p* < 0.01) for the nonfallers. Furthermore, phase-dependent *λ* discriminated as well between fallers and nonfallers as all other gait features combined. The present result suggests that phase-dependent measures of local dynamic stability of daily life walking might be of importance for further development in early fall risk screening tools.

## 1. Introduction

Falls among older people are an important reason for dependence in daily life, reduced quality of life, and admission to hospitals or nursing homes. At a European level, the annual costs of falls among older persons are estimated to be 30 Billion Euro [1]. Early prediction of falls amongst community-dwelling older persons could provide opportunities for early fall prevention. Thus, considerable efforts have been made for early fall risk assessment and fall prediction in older persons.

More than 400 risk factors for falls have been reported (e.g., [2]). Most risk factors have been assessed in laboratory settings or in clinical test situations, and fall risk assessment tools have been developed based on these assessments [3–6]. However, most of these screening tools reflect the performance of the older person at a specific moment in time or they are based on self-report. Furthermore, falls in older persons are often experienced during activities of daily living [7, 8]. Thus, monitoring of behaviour in daily life, rather than assessment by performance tests and self-report, may be important for furthering evidence-based recommendations for fall risk assessment and screening for fall prevention interventions.

Daily life activities, like lying, sitting, standing, and walking, can be identified by body-fixed sensors containing inertial sensors like accelerometers and gyroscopes [9]. Features of the acceleration signal within these activities and in the transition between activities might be important in fall risk assessment [10]. Several measures of gait stability and variability are significantly different between elderly fallers and nonfallers [11–13]. Amongst these is the measure of local dynamic stability that has been suggested to be one of the most sensitive measures of gait instability in older persons [14]. Local dynamic stability *λ* is defined as the rate of exponential increase in infinitely close distances between trajectories in the reconstructed state space of the gait dynamics [15]. These distances are considered as infinitesimal perturbations and, thus, local dynamic stability defines the reaction of the gait dynamics to these perturbations. The gait dynamics are local dynamic stable when *λ* < 0, indicating an exponential decrease in the distance between neighbouring trajectories. In contrast, the gait dynamics are local dynamic unstable when *λ* > 0, indicating an exponential increase in the distance between neighbouring trajectories. Recent extensions of computational methods for local dynamic stability *λ* indicate that *λ* is phase dependent and changes within the gait cycle [16, 17]. Despite promising results, the discriminating ability of phase-dependent *λ* has not been compared to the discriminating abilities of other features of daily life walking.

The main aim of the present study is to compare phase-dependent *λ* with conventional gait features in their ability to discriminate between the daily life walking of community-dwelling elderly fallers and nonfallers.

## 2. Methods

### 2.1. Participants and Data Collection

Inertial sensor data previously studied by Weiss et al. [13] were reanalysed in the present study. The data can be downloaded at http://www.physionet.org/. The data consist of 3 days of 3D-acceleration data from 71 community-dwelling older persons (mean age: 78.36 ± 4.71 yrs; range: 65–87 yrs; gender: 64.79% women; mean height: 1.62 ± 0.07 m; mean weight: 71.98 ± 12.88 kg). None of the participants included had been diagnosed with gait or balance disorders or had cognitive impairments (i.e., Mini Mental State Examination score > 24). The participants were classified as fallers or nonfallers based on retrospective self-report. Participants reporting 2 or more falls in the year prior to testing were considered as fallers; this definition was used to ensure a clear distinction between the two groups and to focus on (multiple) fallers and nonfallers, excluding older adults who may be in an intermediate, less well-defined, and more ambiguous state with respect to their fall history. There was no difference between fallers and nonfallers in age, gender, years of education, height, weight, or body mass index, but a difference in in-lab preferred gait speed (nonfallers: 1.19 ± 0.24 m/s; fallers: 0.97 ± 0.30 m/s). The acceleration along the anterior-posterior (AP), mediolateral (ML), and vertical (V) axes was sampled at 100 Hz by a small inertial sensor (DynaPort Hybrid, McRoberts, The Hague, Netherlands; 87 × 45 × 14 mm, 74 g). The sensor had a range and resolution of ±6 g and ±1 mg, respectively. The acceleration signals were recorded on a Secure Digital (SD) card at a sample frequency of 100 Hz and later transferred to a personal computer for further analysis using Matlab (MathWorks, Natick, MA). The sensor was fitted without any difficulties on a belt on the center of lower back, at the L5 level. The sensor had to be removed during the shower and swimming and occasionally during sleep. The participants received a diary for tracking when and why they took off and put on the device. No specific problems were evident during data collection and retrieval.

### 2.2. Preprocessing of the Data

The classification procedure was restricted to the walking bouts of duration ≥ 60 seconds, identical to those originally analysed by Weiss et al. [13]. This size was chosen to ensure that these were indeed walking segments and that the acceleration derived measures would be robust. The walking bouts were identified by using two filters: one filter was based on the acceleration-magnitude, and the other filter was based on the energy in the frequency domain [13, 18]. The activity bouts were visually observed to ensure that these were indeed valid walking segments. A mean of 28.3 walking bouts (range: 5 to 90) with duration ≥ 60 seconds was identified for each of the participants. There was no significant difference in number of walking bouts between fallers and nonfallers. The reader is referred to Weiss et al. [13] for further details about the participants, protocols, and preprocessing of the 3D-acceleration data.

Intrastep 3D-velocity was estimated from the 3D-acceleration signal. The 3D-acceleration was detrended using an orthogonal wavelet procedure that preserved intrastep variation in the 3D-velocity but removed interstride nonlinear trends [19]. This detrending procedure provides stationary 3D-velocity signal necessary for computation of local dynamic stability [20]. The local maxima of the vertical velocity were defined as the beginning of a step. This step identification method provided similar results to the autocorrelation method used in previous studies based on comparison of the mean step time [12, 21].

### 2.3. State Space Construction Methods

Two 6D state spaces were constructed for each walking bout by the two following methods [20].

**Method 1 .**

Differential coordinate embedding was defined as **x**(*t*) = [*a*_AP_(*t*), *a*_ML_(*t*), *a*_V_(*t*), *v*_AP_(*t*), *v*_ML_(*t*), *v*_V_(*t*)], combining both acceleration signal *a*(*t*) and velocity signal *v*(*t*) in AP, ML, and V directions. The local dynamic stability *λ*_diff_ computed from state space construction Method 1 has the subscript diff in the result section.

**Method 2 .**

Delayed coordinate embedding was defined as **x**(*t*) = [*v*_AP_(*t*), *v*_AP_(*t* + *l*Δ*t*), *v*_ML_(*t*), *v*_ML_(*t* + *l*Δ*t*), *v*_V_(*t*), *v*_V_(*t* + *l*Δ*t*)], where *v*(*t*) is the velocity signal, Δ*t* = 0.01 s is the sampling interval, and *l* is the time lag. This delayed coordinate embedding combines the velocity signal *v*(*t*) in AP, ML, and V directions for the velocity signal and uses a short time lag, *l* = 3, to prevent the blending of phases within the gait cycle. The local dynamic stability *λ*_lag_ computed from state space construction Method 2 has the subscript lag in the result section.

### 2.4. Computation of Phase-Dependent Local Dynamic Stability *λ*

**Figure 1:**
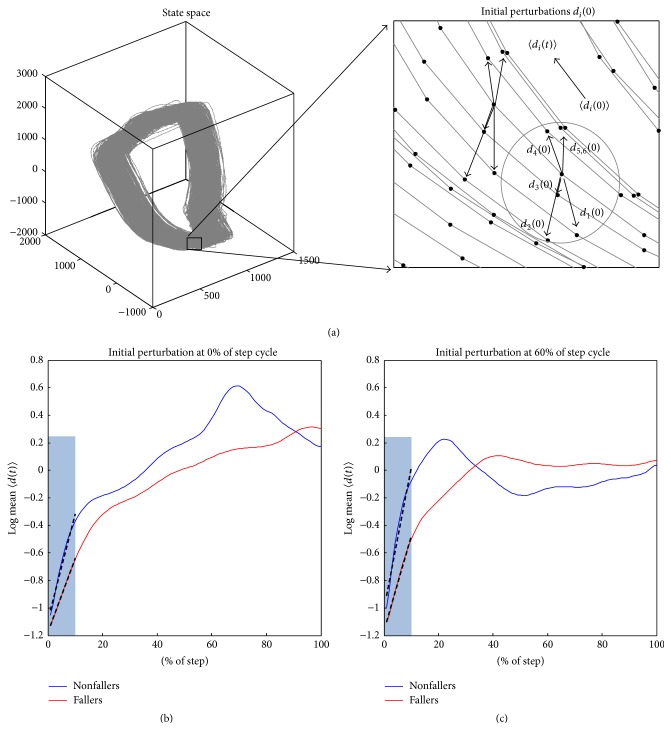
(a) A schematic representation of the reaction distance 〈*d*_*i*_(*t*)〉 based on *i* trajectories within a small neighborhood (gray circle) of the state space. The initial average perturbation distance 〈*d*_*i*_(*t*)〉 was computed from multiple distances *d*_*i*_(*t*) within the neighborhood. Note that the left panel illustrates a 3D state space reconstruction where the computations of 〈*d*_*i*_(*t*)〉 are based on a 6D state space reconstruction. (b) A representative example of a log-reaction curve normalized to the step cycle for a faller (*red*) and a nonfaller (*blue*) for initial perturbation at 0% of the step cycle. (c) The same example of a log-reaction curve for initial perturbation at 60% of the step cycle. The slopes of the regression lines for the initial 10% (black lines in shaded areas of (a) and (b)) were defined as the local dynamic stability according to (1).

Phase-dependent local dynamic stability was defined according to a method developed by Ihlen et al. [16] and based on two equations:

(1) λ=ln⁡ditstept,

(2) λ=1tnln⁡ditnstepdi0step,

where 〈*d*_*i*_(*t*)〉 is the reaction curve of the initial perturbation 〈*d*_*i*_(0)〉 and the outer brackets 〈⋯〉 are the mean across all steps in the walking bout. The initial perturbation was defined as the distance *d*_*i*_(0) between the reference point and the *i*th neighbourhood trajectory within a small neighbourhood of predefined size (see Figure 1(a)). The initial perturbation was considered at 0%, 20%, 40%, 60%, and 80% of the step cycle (see Figures 1(b) and 1(c), e.g., for 0% and 60%). The reaction distance *d*_*i*_(*t*) of the initial distance *d*_*i*_(0) was traced to the next starting point of a step. The average reaction distance 〈*d*_*i*_(*t*)〉 was computed across all *i*th neighbourhood trajectories. The reaction distance, 〈*d*_*i*_(*t*)〉, for less than 10 neighbourhood trajectories or with instantaneous stride time outside the 5% and 95% percentiles was excluded for further analysis. The portion of excluded 〈*d*_*i*_(*t*)〉 was less than 10% of the total number of strides for all participants and these strides were typically short periods of deviating patterns of the acceleration and velocity signals due to large deviations from normal patterns. In (1), the remaining 〈*d*_*i*_(*t*)〉 was normalized to step time before *λ* was assessed as the linear regression slope for the first 10% of the step cycle (see Figure 1(b)). In (2), the remaining 〈*d*_*i*_(*t*)〉 was not normalized and 〈*d*_*i*_(*t*)〉 was the reaction distance at time *t*_*n*_ equal to 10% of the step cycle. The first 10% of the step cycle was considered to prevent influence of curvatures in state space trajectories [22]. The median of *λ* was computed across all walking bouts for each participant. In addition, the conventional phase-independent *λ*_wolf_ was computed by the method of Wolf et al. [23]. Wolf's method was applied to the acceleration signal in the AP, ML, and V directions, separately. A 6D delayed coordinate embedding was used with time lag, *l* = 8, which was the mean lag for the first minima of the average mutual information function [24]. The Matlab code for the phase-dependent measures, *λ*_diff_ and *λ*_lag_, is available at http://www.physionet.org/.

### 2.5. Test-Retest Reliability of Phase-Dependent Local Dynamic Stability *λ*

Different circumstances of walking, like turns, walking surfaces, obstacles, variations in walking speed, and dual tasking, may introduce random fluctuations in phase-dependent local dynamic stability *λ* between walking bouts. Even though the median of each selected feature across several walking bouts will reduce these fluctuations, it is uncertain if the median has sufficient reliability across walking bouts within a 3-day period. Thus, the test-retest reliability of the median of local dynamic stability *λ* was assessed by interclass correlation (ICC) absolute agreement for the first and the last 1/3 of the walking bouts within the 3-day recording period.

### 2.6. Partial Linear Square Discriminant Analysis (PLS-DA)

**Figure 2:**
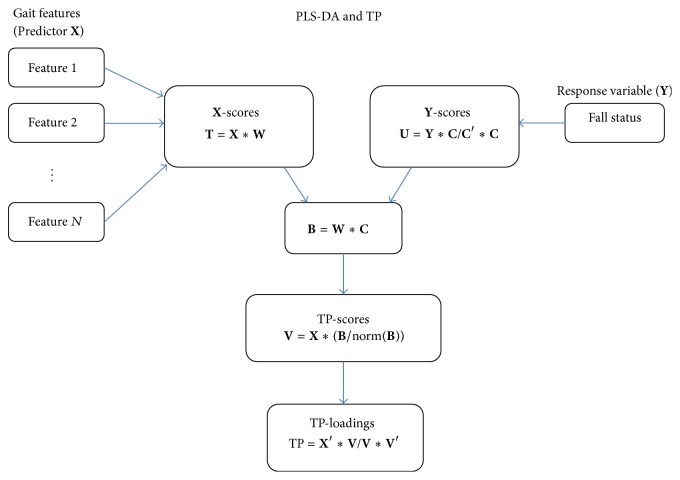
A schematic illustration of partial least square discriminant analysis (PLS-DA) and target projection (TP) used in the present study. The prediction matrix **X** has a number of columns equal to the number of gait features and number of rows equal to the number of participants. The high number of gait features is projected to a small number of principle axes in the feature space and this projection is defined as the **X**-scores (**T**). The projection is provided by a weight matrix **W** for the gait feature matrix **X** and by a weight matrix **C** for the categorical (faller and nonfaller) response variable **Y**. The target projection method combines the weight matrices **W** and **C** in order to define the influence of each gait feature in the discrimination between fallers and nonfallers. The cross-product **B** of weights **W** and **C** is used to calculate the target projection scores **V**, which is a variable containing one score for each older person which maximizes the discrimination between fallers and nonfallers. The target projection score **V** is used to define the target projection loadings TP containing one loading for each gait feature that denotes its influence on the target projection score.

PLS-DA relates the predictor matrix **X** of gait features with the response **Y** of fall status (see Figure 2). PLS-DA is able to identify a low-dimensional latent structure (**T**) from a large number of gait features **X** which discriminates between fallers and nonfallers. In contrast to other regression approaches, PLS-DA is designed to perform discriminate analyses based on a large set of noisy and collinear predictors **X** and is therefore suitable for the large number of gait features investigated in the present study. The present study used a nonlinear iterative partial least square (NIPALS) algorithm extended by a target projection (TP) as summarized in Figure 2 [25–27]. The TP-loadings define the contribution of each gait feature in the PLS model. A TP-loading closer to −1 or 1 indicates that the gait feature has a strong influence in discriminating between fallers and nonfallers, whereas a TP-loading close to 0 indicates that the feature has little or no influence in discriminate analyses. Thus, the TP-loading provides a ranking list of the most influential gait features for the classification of fallers and nonfallers. Three different predictor matrices **X** were defined for the PLS-DA to compare the discrimination ability of phase-dependent local dynamic stability with other gait features (see Table 1). All gait features in the predictor matrices **X** were converted to *z*-scores before the application of PLS-DA. A PLS-DA cross-validation procedure was used to estimate how well the model would generalize to new samples from the same population [28]. Four latent variables provided the minimum error of the cross-validation for all predictor matrices **X** and were used in the PLS-DA. Sensitivity and specificity and area under the ROC curve (AUC) were defined based on the real and predicted outcome variables from PLS-DA. All analyses were performed in Matlab R2014a.

## 3. Results

**Figure 3:**
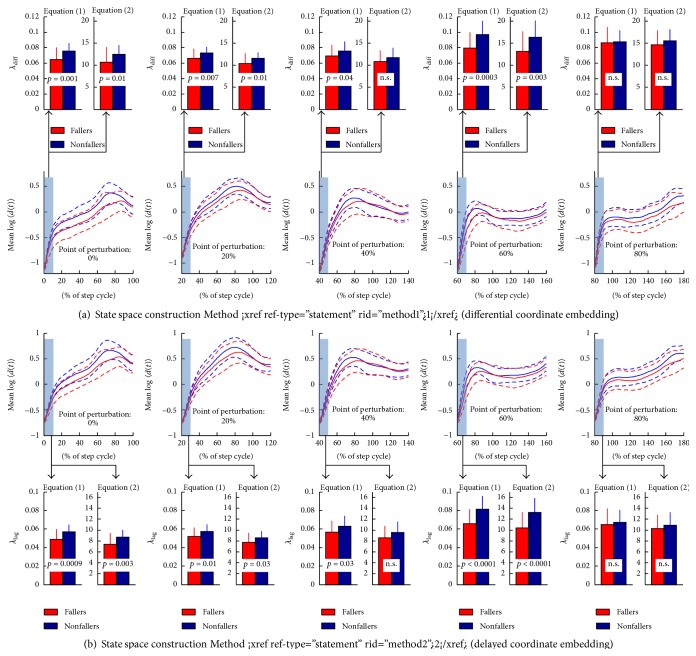
(a) Mean ± 1SD of the log-median 〈*d*_*i*_(*t*)〉 for fallers (*red*) and nonfallers (*blue*) defined by the state space reconstruction Method 1 (differential coordinate embedding) for initial perturbation at 0%, 20%, 40%, 60%, and 80% of the step cycle. The smaller upper subplots show mean ± 1SD of local dynamic stability *λ*_diff_ for fallers (*red*) and nonfallers (*blue*) together with *p* values. (b) Mean ± 1SD of the log-median 〈*d*_*i*_(*t*)〉 for fallers (*red*) and nonfallers (*blue*) defined by the state space reconstruction Method 2 (delay coordinate embedding) for initial perturbation at 0%, 20%, 40%, 60%, and 80% of the step cycle. The smaller upper subplots show mean ± 1SD of local dynamic stability *λ*_lag_ for fallers (*red*) and nonfallers (*blue*) together with *p* values.

**Figure 4:**
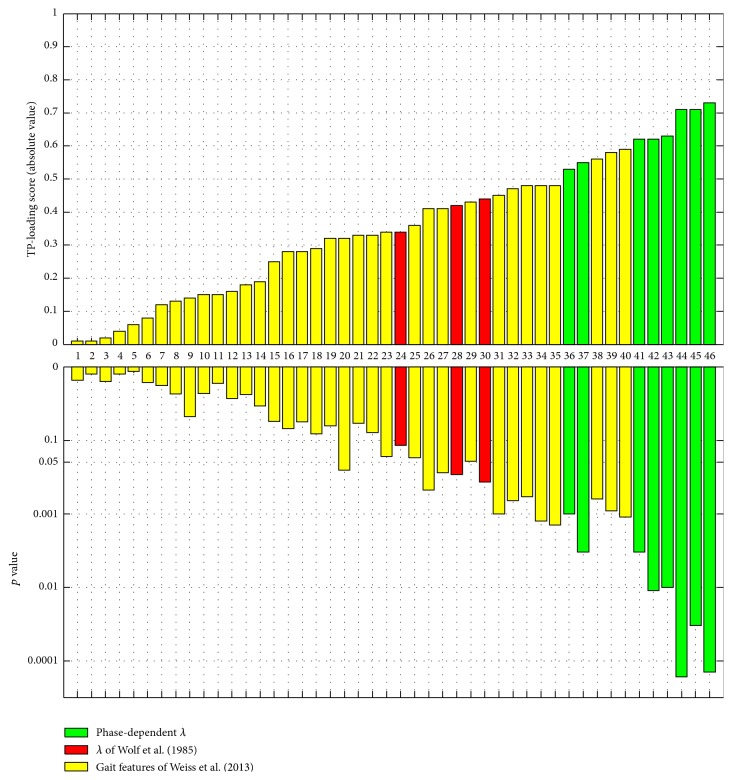
The TP-loading scores and corresponding *p* values for 46 gait features (predictor matrix **X**_1_ in Table 1). The feature numbers in the middle are linked to the list of gait features as follows: (1) total number of walking bouts, (2) ML harmonic ratio, (3) total percent of walking duration, (4) V step symmetry, (5) AP amplitude of dominant frequency, (6) ML step regularity, (7) V acceleration root-mean-square, (8) ML acceleration root-mean-square, (9) total number of steps, (10) V harmonic ratio, (11) ML step symmetry, (12) AP acceleration root-mean-square, (13) AP slope of dominant frequency, (14) AP stride regularity, (15) ML stride regularity, (16) V step regularity, (17) ML acceleration range, (18) V stride regularity, (19) AP step symmetry, (20) median walking bout duration, (21) V acceleration range, (22) AP harmonic ratio, (23) ML width of dominant frequency, (24) V *λ*_wolf_, (25) AP step regularity, (26) V slope of dominant frequency, (27) V width of dominant frequency, (28) ML *λ*_wolf_, (29) AP width of dominant frequency, (30) AP *λ*_wolf_, (31) V amplitude of dominant frequency, (32) ML amplitude of dominant frequency, (33) ML slope of dominant frequency, (34) median number of steps for bout, (35) AP acceleration range, (36) *λ*_diff_ (phase: 0%, (2)), (37) *λ*_lag_ (phase: 0%, (2)), (38) Cadence, (39) average stride duration, (40) average step duration, (41) *λ*_diff_ (phase: 60%, (2)), (42) *λ*_lag_ (phase: 0%, (1)), (43) *λ*_diff_ (phase: 0%, (1)), (44) *λ*_lag_ (phase: 60%, (1)), (45) *λ*_diff_ (phase: 60%, (1)), and (46) *λ*_lag_ (phase: 60%, (2)). The phase-dependent local dynamic stability measures, *λ*_lag_ and *λ*_diff_, are represented as* green bars* whereas conventional local dynamic stability measures, *λ*_wolf_, are represented as* red bars*. The yellow bars represent gait features used in Weiss et al. (2013).

**Figure 5:**
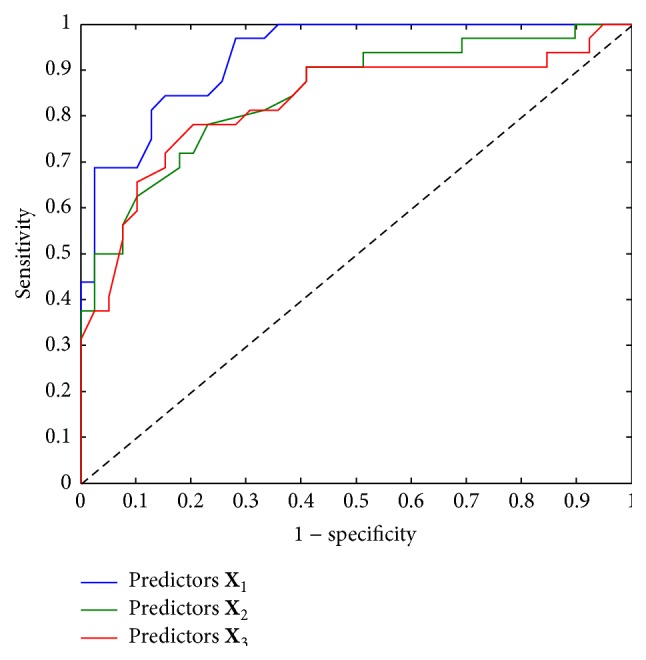
ROC curves summarizing the performance of the PLS-DA for the predictor matrix **X**_1_ (*blue*), predictor matrix **X**_2_ (*green*), and predictor matrix **X**_3_ (*red*) of gait features (see Table 1 for their definitions).

Figure 3 shows the mean reaction curve of the fallers and nonfallers for initial perturbation at 0%, 20%, 40%, 60%, and 80% of the step cycle. The figure indicates that the reaction distance curve for both elderly fallers and nonfallers has a phase-dependent shape. The nonfallers had a significantly larger local dynamic stability, median *λ*, compared to the fallers at 0%, 20%, and 60% of the step cycle, irrespective of the state space reconstruction method and definition of *λ* (see upper and lower panels in Figure 3). The TP-loadings of all 46 included gait features indicate that phase-dependent *λ* at 0 and 60% of step cycle was most influential in discriminating between elderly fallers and nonfallers (see green bars in Figure 4). The conventional measures of local dynamic stability had less influence in the discrimination analysis compared to the phase-dependent *λ* (compare red bars of *λ*_wolf_ with green bars of *λ*_lag_ and *λ*_diff_ in Figure 4). Increased error, decreased specificity, and decreased AUC were found when the phase-dependent measures, *λ*_diff_ and *λ*_lag_, were removed (see Table 2 and Figure 5). Furthermore, the eight phase-dependent measures, *λ*_diff_ and *λ*_lag_, performed as good as all the 38 gait features together in classification of fallers and nonfallers (see third column in Table 2 and the red ROC curve in Figure 5). In addition, all the phase-dependent measures, median *λ*_diff_ and *λ*_lag_, included in the discriminate analysis had high test-retest reliability (ICC coefficients > 0.80; see Table 3). Thus, phase-dependent local dynamic stability seems to be an important feature for the classification of fallers and nonfallers in community-dwelling older persons.

## 4. Discussion

The main purpose of the present study was to compare phase-dependent local dynamic stability measures with more conventional gait features in their ability to discriminate between community-dwelling elderly fallers and nonfallers. The phase-dependent *λ*_diff_ and *λ*_lag_ at 0% and 60% of the step cycle had the best classification performance and considerably improved the PLS-DA, compared to the 38 conventional features of daily life walking.

In a treadmill study, Ihlen et al. [17] found that healthy older persons had larger phase-dependent *λ*_diff_ and *λ*_lag_ compared to young adults. In contrast, in the present study, the phase-dependent *λ*_diff_ and *λ*_lag_ were larger in older nonfallers compared to fallers. These contrasting findings may be due to several reasons. First, a recent study indicates that gait characteristics obtained in in-lab studies are different from those recorded for daily life walking [29]. Thus, the more unstable gait dynamics of elderly nonfallers might be due to a more heterogeneous and challenging walking environment for this group including more frequent turns and multitasking while walking. These factors might contribute to the larger *λ*_diff_ and *λ*_lag_ compared to the fallers but might also indirectly reflect subtle decline in balance and mobility in the fallers group. Second, the increased local dynamic stability found for the fallers might also reflect adaption in the gait dynamics towards more cautious gait including less frequent turns and multitasking while walking and less challenging walking environment. However, the present study cannot conclude whether the larger *λ*_diff_ and *λ*_lag_ in the nonfallers are due to differences in external environmental factors or internal neuromuscular factors or a combination of these two. Further studies are needed to include contextual information of daily life walking in community-dwelling older persons.

Rispens et al. [12] found that local dynamic stability, *λ*_wolf_, computed by the method of Wolf et al. [23] for the acceleration signal in the V direction was able to discriminate between fallers and nonfallers. However, in the present study, the phase-dependent *λ*_diff_ and *λ*_lag_ were found to be more sensitive to falls status of community-dwelling older person compared to *λ*_wolf_. In contrast to the findings of Rispens et al. [12], *λ*_wolf_ did not influence the classification of fallers and nonfallers in the present study.

The present study also shows the potential of PLS-DA for the comparison of the influence of different gait features to discriminate between fallers and nonfallers. Numerous features of gait stability and variability have been introduced in the last decades, but their abilities to discriminate between fallers and nonfallers are seldom compared [11]. The TP-loadings in Table 2 are able to rank the influence of the different gait features in the classification of elderly fallers and nonfallers. Table 2 indicates that many of these features have significantly different mean values for fallers and nonfallers, while the discriminatory power is low (i.e., TP-loading < 0.5). A consensus on a procedure to compare the abilities of different gait features in the classification of elderly fallers and nonfallers, like PLS-DA, might have important value for evaluation of new features of gait stability and variability. Furthermore, procedures like PLS-DA might also be helpful for the identification of fall risk profiles for different groups of older people, and the procedures might be extended to include clinical test scores and demographic variables.

The present study has several limitations. First, the present study did only distinguish between fallers and nonfallers based on retrospective fall reports from a relatively small sample of community-dwelling older adults. The specificity, sensitivity, and AUC reported in the present study are in the upper end of values that could be expected from a perfect fall prediction model [30]. Thus, it is likely that specificity, sensitivity, and AUC will decrease for *λ*_diff_ and *λ*_lag_ in a prediction model of prospective falls. Consequently, further studies on larger samples with prospective fall data are necessary before concluding that phase-dependent *λ*_diff_ and *λ*_lag_ will improve fall prediction models or early fall risk assessment in the population of community-dwelling older adults.

Second, demographic variables and variables of clinical tests used for fall risk assessment, like tests of balance and mobility performance, were not included in the classification procedure. Inertial sensor based tools for unsupervised in-home test of physical function, including mobility and balance, could also contribute to the improvement of early fall risk assessment in community-dwelling older adults [31]. However, former studies have shown that features of daily life walking improve the risk assessment when combined with instrumented tests of mobility performance [10, 13]. Nevertheless, falls have multifactorial causes including medication, urinary control, vision, footwear, environmental hazards, cognitive function, mental health, and fear of falling, to mention but a few, and it is therefore likely that a combination of outcomes of clinical tests and features of daily life activities will optimize fall risk assessment and fall prediction models. Even though the inclusion of *λ*_diff_ and *λ*_lag_ might further improve the fall risk assessment when combined with clinical tests, issues like cost (and maintenance cost) of accelerometers, unsupervised device handling in an in-home setting, provision and retrieval from patient in a clinical setting, and the potential for an easy-to-use online estimation of *λ*_diff_ and *λ*_lag_ will decide the feasibility of the use of *λ*_diff_ and *λ*_lag_ in fall risk assessment tools. Thus, further studies and cost-benefit analyses have to be conducted to determine the usability and feasibility of these analyses when implemented in smartphone and desktop application and the gain in accuracy of fall risk assessment needed to compensate potential decline in clinical feasibility.

Third, the relationship between phase-dependent stability *λ*_diff_ and *λ*_lag_ and variables related to the health status of the older adults was not investigated. Investigation of these relationships would be important to improve the clinical interpretation of *λ*_diff_ and *λ*_lag_ and thus should be included in further studies. In addition, assessment of *λ*_diff_ and *λ*_lag_ could be combined with experimental in-lab research of stability, like experimental perturbation studies of in-lab gait, as well as studies on neurophysiological mechanisms in animal models to improve the understanding of the underlying mechanisms of *λ*_diff_ and *λ*_lag_ [32, 33].

Fourth, the time consumption of the computational steps (i.e., gait bout identification, preprocessing, and estimation procedure) to assess *λ*_diff_ and *λ*_lag_ was not recorded. The time consumption of these steps would be important to decide the possibility for online computation of *λ*_diff_ and *λ*_lag_ which is necessary for clinical feasible smartphone and desktop application.

Fifth, even if the present study did investigate the test-retest reliability of *λ*_diff_ and *λ*_lag_ based on the first and the last 1/3 of the walking bouts during the 3-day recording, this is a considerable shorter test-retest interval compared to the 1-week test-retest interval considered in Rispens et al. [12]. ICC for 1-week test-retest might be weaker compared to the ICC found in the present study and further studies should investigate test-retest reliability of the local divergence features for longer test-retest intervals.

Sixth, the accuracy of the phase-dependent *λ*_diff_ and *λ*_lag_ is dependent on the reliability of the step identification. The inertial sensor was placed on the lower back which makes heel strike and toe-off events more difficult to identify within the gait cycle. Thus, the phase-dependent *λ*_diff_ and *λ*_lag_ in Figure 3 were not defined according to single and double support phases within the gait cycle, but according to the local peaks of the vertical velocity. The employment of advanced step identification algorithms might define the phase-dependent *λ*_diff_ and *λ*_lag_ according to heel strike and toe-offs, but further validation of these algorithms is necessary [34]. Furthermore, as inertial sensors become smaller and more wearable, further studies should include an additional sensor on the lower extremities and/or insole data to identify heel strikes and toe-offs and thereby single and double support phases.

Seventh, the sample size used in the present study is small. Rispens et al. [12] were not able to replicate the results of Weiss et al. [13] for some of the spectral features for another study with a larger sample size. A similar contrast in results might be present for phase-dependent local dynamic stability when replicated for different groups of community-dwelling older persons. Thus, further studies should replicate these initial findings on cohorts of community-dwelling older persons with different health status. Finally, the present study suggests that phase-dependent *λ*_diff_ and *λ*_lag_ are related to falls status and might be important to include in fall risk assessments and fall prediction models. Several studies indicate that measures of gait stability and variability improve fall risk assessment and fall prediction models when compared to assessments and models based on clinical tests and fall history [12, 13]. Thus, further application of phase-dependent *λ*_diff_ and *λ*_lag_, to track changes in falls status and to prospectively identify fallers, is needed to determine their influence in fall risk assessments and fall prediction models.

## 5. Conclusions

The present study compared phase-dependent measures of local dynamic stability of elderly fallers and nonfallers in daily life walking with existing features of gait stability and variability. These phase-dependent measures had the best classification performance of all included gait features and improved the discrimination between elderly fallers and nonfallers compared to all other features of daily life walking. Thus, phase-dependent measures of local dynamic stability might be of importance for further development in early fall risk assessment, fall prediction, and fall prevention amongst community-dwelling older persons. The present results set the stage for follow-up prospective studies in larger cohorts and clinical feasibility studies to further assess the potential of these metrics.

## Figures and Tables

**Table 1:** The gait features contained in the three predictor matrices **X** used in the partial least square discriminatory analysis (PLS-DA) of elderly fallers and nonfallers. The gait features written in italic style are the same features used in Weiss et al. (2013) [13].

Predictor matrix **X**_1_ (46 gait features)	Predictor matrix **X**_2_ (38 gait features)	Predictor matrix **X**_3_ (8 gait features)
*λ*_diff_ (phase: 0%, (1))	—	*λ*_diff_ (phase: 0%, (1))
*λ*_diff_ (phase: 0%, (2))	—	*λ*_diff_ (phase: 0%, (2))
*λ*_diff_ (phase: 60%, (1))	—	*λ*_diff_ (phase: 60%, (1))
*λ*_diff_ (phase: 60%, (2))	—	*λ*_diff_ (phase: 60%, (2))
*λ*_lag_ (phase: 0%, (1))	—	*λ*_lag_ (phase: 0%, (1))
*λ*_lag_ (phase: 0%, (2))	—	*λ*_lag_ (phase: 0%, (2))
*λ*_lag_ (phase: 60%, (1))	—	*λ*_lag_ (phase: 60%, (1))
*λ*_lag_ (phase: 60%, (2))	—	*λ*_lag_ (phase: 60%, (2))
*λ*_wolf_^*∗*^	*λ*_wolf_^*∗*^	—
*Acceleration range* ^*∗*^	*Acceleration range* ^*∗*^	—
*Acceleration root-mean-square* ^*∗*^	*Acceleration root-mean-square* ^*∗*^	—
*Amplitude of dominant frequency* ^*∗*^	*Amplitude of dominant frequency* ^*∗*^	—
*Average stride duration*	*Average step duration*	—
*Average step duration*	*Average step duration*	—
*Cadence*	*Cadence*	—
*Harmonic ratio* ^*∗*^	*Harmonic ratio* ^*∗*^	—
*Median walking bout duration*	*Median walking bout duration*	—
*Median number of steps for bout*	*Median number of steps for bout*	—
*Slope of dominant frequency* ^*∗*^	*Slope of dominant frequency* ^*∗*^	—
*Step symmetry* ^*∗*^	*Step symmetry* ^*∗*^	—
*Step regularity* ^*∗*^	*Step regularity* ^*∗*^	—
*Stride regularity* ^*∗*^	*Stride regularity* ^*∗*^	—
*Total number of steps*	*Total number of steps*	—
*Total number of walking bouts*	*Total number of walking bouts*	—
*Total percent of walking duration*	*Total percent of walking duration*	—
*Width of dominant frequency* ^*∗*^	*Width of dominant frequency* ^*∗*^	—

^*∗*^Gait feature defined for AP, ML, and V direction, separately.

**Table 2:** Classification performance for predictor matrices **X**_1_, **X**_2_, and **X**_3_ (see Table 1 for their definitions).

	Predictors **X**_1_	Predictors **X**_2_	Predictors **X**_3_
Sensitivity	0.72	0.72	0.69
Specificity	0.90	0.79	0.87
AUC	0.93	0.84	0.83
Error (1 – accuracy)	0.18	0.24	0.21

**Table 3:** Interclass correlation (ICC) coefficient and its 95% confidence interval (CI) for the phase-dependent local dynamic stability measures, *λ*_lag_ and *λ*_diff_.

Features	ICC	ICC (95% CI)
*λ*_diff_ (phase: 0%, (1))	0.90	[0.84, 0.94]
*λ*_diff_ (phase: 0%, (2))	0.89	[0.82, 0.93]
*λ*_diff_ (phase: 60%, (1))	0.92	[0.88, 0.95]
*λ*_diff_ (phase: 60%, (2))	0.90	[0.85, 0.94]
*λ*_lag_ (phase: 0%, (1))	0.86	[0.77, 0.91]
*λ*_lag_ (phase: 0%, (2))	0.85	[0.76, 0.91]
*λ*_lag_ (phase: 60%, (1))	0.93	[0.88, 0.95]
*λ*_lag_ (phase: 60%, (2))	0.92	[0.87, 0.95]
